# Evaluating food safety in China: regional disparities and dynamic evolution

**DOI:** 10.3389/fpubh.2026.1821428

**Published:** 2026-06-18

**Authors:** Tong Zhao, Jingjing Wang, Yun Luo, Dan Liu, Linlin Zhang

**Affiliations:** 1 College of Rural Revitalization, Anhui Agricultural University, Hefei, Anhui, China; 2 College of Economics and Management, Nanjing Agricultural University, Nanjing, Jiangsu, China; 3 College of Business Administration, Anhui University of Finance and Economics, Bengbu, Anhui, China

**Keywords:** food safety, food safety performance, kernel density, regional disparities, stakeholder theory

## Abstract

**Background:**

Food safety remains a critical global concern, and assessing regional food safety performance is essential for effective policy design. However, systematic evidence on regional disparities and their evolution in China remains limited.

**Methods:**

Grounded in stakeholder theory, this study constructs a multidimensional framework encompassing producers, government regulation, and social harm. Using panel data from 30 Chinese provinces (2016–2021), food safety performance is measured using the entropy-weighted TOPSIS method, while regional disparities and their dynamics are analyzed through Dagum Gini decomposition and kernel density estimation.

**Results:**

Food safety performance exhibits a steady upward trend, with the composite index increasing from 0.070 to 0.189. The spatial pattern follows “Eastern > Western > Northeastern > Central,” with the Central region lagging behind. Regional disparities have widened over time, driven primarily by inter-regional differences. Kernel density results indicate overall improvement accompanied by increasing dispersion, with weakening polarization in the Eastern and Western regions but a contrasting trend in the Central region.

**Conclusion:**

Although food safety performance in China has improved, significant regional disparities persist. More targeted and region-specific policy interventions are needed to enhance governance effectiveness.

## 1 Introduction

China's sustained socioeconomic progress has gradually shifted residents' dietary priorities from the subsistence imperative of “eating enough” to the higher-order aspiration of “eating well,” marked by nutritional balance and superior quality. This transition has redefined the central challenge confronting China's food industry—from assuring adequate supply to safeguarding food quality and safety (1). Across both emerging and developed economies, rising incomes, urbanization, and the globalization of food supply chains have intensified public concern over food safety and quality standards (2). While the sector's rapid expansion has met consumers' growing appetite for dietary diversity, the ongoing modernization of agriculture has also made food supply chains increasingly complex. This, in turn, has heightened the intricacy and uncertainty of food-safety risks (3). In recent years, high-profile incidents—including recycled “gutter oil,” melamine-tainted infant formula, and “pit-fermented” pickled vegetables—have underscored that food safety has become a recurring, rather than episodic, crisis. Such repeated failures not only endanger consumer health but also undermine public trust and disrupt market order. When they occur at scale, they may further trigger public-health emergencies and threaten social stability (4).

It is important to clarify that this study focuses on food safety rather than food security. Food safety, as defined by the World Health Organization (WHO), refers to the assurance that food does not cause harm to consumers during preparation, processing, storage, and distribution. It encompasses hazards such as chemical contaminants, pathogenic microorganisms, and adulterants. In contrast, food security, as defined by the Food and Agriculture Organization (FAO), refers to the situation in which all people, at all times, have physical, social, and economic access to sufficient, safe, and nutritious food that meets their dietary needs and preferences. Accordingly, this study examines food safety performance across multiple dimensions, focusing on producers, government regulation, and social harm.

To address the increasingly complex challenges of food safety, China has continuously explored and refined its approach along two dimensions: the legal-institutional framework and the regulatory governance system. At the legal level, China has amended the Food Safety Law multiple times, thereby dynamically optimizing institutional design and regulatory requirements to establish a legal regime that responds to newly emerging food-safety problems (5). Similar regulatory reforms have also been implemented internationally. For example, the European Union has strengthened its integrated food-chain governance through the General Food Law framework, while the United States has promoted preventive food-safety management under the Food Safety Modernization Act (FSMA). These reforms reflect a broader international shift from reactive regulation toward risk-based and preventive governance approaches. At the governance level, guided by the “farm-to-table” continuum, China has clarified the boundaries of multi-agency responsibilities and established coordination mechanisms. Through a closed-loop and collaborative model—including risk detection through random inspections, source tracing, and a division-of-labor response system—China has sought to implement end-to-end supervision across all stages of food production and circulation (6). However, existing evidence indicates that despite substantial regulatory efforts by the Chinese government, overall food-safety performance has not improved commensurately (7). One important reason is the insufficient recognition of regional disparities in food safety, which has led to a failure to adopt strategies tailored to local conditions (8). Accordingly, rigorous food-safety evaluation can provide policy-relevant insights to support targeted regional development and the further strengthening of governance capacity.

A growing body of research has undertaken quantitative analyses of food safety, seeking to define its scope as comprehensively and rigorously as possible and to develop indicator systems that are both robust and practically implementable. International studies have evaluated food safety from multiple perspectives, including institutional quality, agricultural production conditions, supply-chain traceability, public-health outcomes, and consumer risk perception (9). According to the extant literature, indicators used to measure food-safety performance or risks in China can be broadly classified into three categories. The first category concerns producers' certification behaviors, including QS certification and HACCP certification (10). As an effective signaling mechanism, certification schemes are widely adopted worldwide as a key policy instrument for safeguarding food safety (11). However, many studies argue that China's certification market is dominated by official certification bodies, which, due to pronounced principal–agent problems, are vulnerable to regulatory capture by producers. This claim is supported by repeated exposures of “certified” products whose production processes fail to meet relevant standards, as well as substantial gaps between certification outcomes and actual product quality (12). Conversely, other studies suggest that for products with technologically complex production and processing procedures—such as meat products—the implementation of HACCP systems can more substantially enhance product quality (6).

The second category comprises indicators of government regulation. In China, the government primarily relies on the pass rate of supervisory sampling inspections to assess food-safety performance (36). The chief advantages of this measure are its simplicity, intuitive interpretability, and relatively low cost of data acquisition. Moreover, in routine regulatory practice, market supervision authorities can readily operationalize it as the proportion of qualified inspection batches relative to the total number of sampled batches. However, its limitations are equally apparent in at least two respects. First, given the vast diversity of food products and the complexity of regulatory targets in China, sampling inspections may suffer from limited coverage and inadequate representativeness. This, in turn, constrains the credibility of the resulting data (13, 14). Second, because inspection pass rates are often tied to local governments' performance evaluations, the incentive structure can substantially weaken the efficiency of problem-oriented regulation (15). Accordingly, indicators of governmental oversight can capture only part of the overall food-safety picture. The third category focuses on food-safety incidents reported by the media. The number of food-safety incidents (FSI) constitutes an important metric for gauging food-safety risk. Systematic classification and tabulation of media-exposed incidents can help identify areas where problems are concentrated and pinpoint key risk targets (16). Yet this class of indicators is also imperfect. Incident counts are readily shaped by media preferences and reporting biases, and the frequency of exposure does not necessarily coincide with underlying safety performance (17).

Overall, single dimensions and single indicators each entail distinct strengths and inherent limitations. Reliance on any single measure—or even a single class of measures—makes it difficult to fully depict the landscape of food-safety problems in a comprehensive, accurate, and objective manner. It also limits the ability to identify deeper structural drivers and to address the complexity of food systems systematically. Moreover, existing international studies have largely focused either on specific food-safety incidents or on isolated dimensions of governance, while comparatively limited attention has been paid to comprehensive regional assessments integrating multiple stakeholders and dynamic spatial disparities, especially in large developing countries. Accordingly, this study pursues four objectives: (1) to develop a food-safety evaluation index system based on a stakeholder-theory framework; (2) to characterize the spatial distribution of food-safety performance across 30 Chinese provinces; (3) to decompose the sources of regional disparities in food-safety performance; and (4) to trace the dynamic evolution of the distribution of food-safety performance over time.

## 2 Multidimensional evaluation framework

Developing sound, scientifically grounded standards for food-safety assessment is essential for promoting the sustainable development of the food industry. A substantial body of research indicates that government action alone cannot comprehensively cover all dimensions of food safety; instead, effective governance requires the integration of key stakeholders—particularly producers and consumers—into participatory, collaborative arrangements to improve both the quality and efficiency of food-safety performance (18). Accordingly, grounded in stakeholder theory, this study proposes an assessment framework structured around three core dimensions: (i) upstream prevention and control by producers, (ii) governmental regulatory intervention, and (iii) the societal harm borne by consumers. As summarized in Table 1, the framework is operationalized as a multi-level evaluation index system consisting of three dimensions, six sub-indices, and ten specific indicators.

**Table 1:** Food safety evaluation index system.

Objective	Dimension	Index	Variable	Description of variable (unit)	Attribute
Food safety assessment	Food-safety behavior of producers (Producer level)	Proactive quality control	Producer certification behavior	Number of newly HACCP-certified food producers/Total number of food producers	+
		Passive inspection enforcement	Producer violation behavior	Number of food producers with violations/Total number of food producers	–
	Government food-safety regulation (Government level)	Regulatory input	Inspection intensity per capita	Number of food inspection batches/Year-end population	+
			Inspection intensity per producer	Number of food inspection batches/Total number of food producers	+
			Supervised inspection efficiency	Supervised inspection failure rate/Random inspection failure rate	+
		Regulatory output	Supervised inspection failure rate	Number of non-compliant food samples/Total number of inspected food samples	–
		Regulatory outcomes	Random inspection failure rate	Compliant food samples/Total number of food producers	–
	Food-safety social harm (Consumer level)	Degree of social harm	Number of food safety incidents	Number of foodborne disease incidents/GDP	–
			Number of food safety illness cases	Number of foodborne disease cases/Year-end population	–

### 2.1 Food-safety behavior of producers

Producers are directly involved in all stages of food production, and their actions span nearly every node of the production chain; consequently, they constitute the primary upstream source of food-safety risk (19). Accordingly, this study conceptualizes producers' food-safety behavior along two complementary dimensions: proactive quality control and passive inspection enforcement, so as to capture both ex ante preventive efforts and ex post compliance outcomes. These two dimensions are operationalized, respectively, through producer certification behavior and producer violation behavior. First, proactive quality control is reflected in producer certification behavior. This is measured as the ratio of newly HACCP-certified food producers to the total number of food producers. Within such systems, the HACCP framework is internationally recognized as a benchmark approach to risk management and a key criterion for evaluating food-safety quality-control capacity (20). However, certification may not always accurately reflect actual food-safety performance due to potential certification distortion or symbolic compliance. Therefore, in this study, producer certification behavior is interpreted as an indicator of formalized proactive quality control efforts rather than realized safety outcomes. Second, passive inspection enforcement is captured by producer violation behavior, which is measured by the ratio of food producers with violations to the total number of food producers. Non-compliant production refers to the manufacture of foods that contravene food-safety laws and regulations, including the illicit addition of harmful substances, excessive heavy-metal residues, misuse of food additives, failure to meet quality standards, and microbial contamination (7). While this indicator provides important information for assessing food-safety performance, it is also influenced by regulatory intensity, as higher inspection frequency increases the likelihood of detecting violations. Therefore, the observed violation rate reflects not only underlying compliance behavior but also the degree of regulatory exposure.

### 2.2 Government food-safety regulation

As the central actor in public governance, government plays an indispensable role in food-safety regulation. Establishing multidimensional, end-to-end regulatory measures is a critical means of remedying market failures and safeguarding food safety. Although the literature has examined food-safety regulation extensively, scholars have adopted differing logics and emphases when constructing regulatory-indicator frameworks. Nonetheless, most studies converge on three fundamental dimensions: regulatory input, regulatory output, and regulatory outcomes (6, 21). Building on these insights, and to more clearly distinguish between regulatory input (effort), regulatory output (inspection results), and regulatory outcomes (actual food-safety conditions), this study operationalizes government regulation using three analytically distinct dimensions.

First, regulatory input reflects the level of resources and enforcement effort devoted by the government. It is measured by inspection intensity per capita and inspection intensity per producer, which capture the scale and coverage of regulatory activities. In addition, supervised inspection efficiency is incorporated as a complementary indicator of regulatory input, defined as the ratio of the supervised inspection failure rate to the random inspection failure rate. This indicator reflects the extent to which regulatory efforts are effectively targeted toward higher-risk entities, thereby capturing the allocative efficiency of regulatory resource allocation. Detailed measurement principles are provided in Appendix 1. Second, regulatory output refers to the direct results of regulatory enforcement, namely the extent to which violations are detected through inspections. This study measures regulatory output using the supervised inspection failure rate, defined as the number of non-compliant food samples divided by the total number of inspected samples. Because supervised inspections are typically risk-oriented and problem-targeted, a higher failure rate indicates stronger effectiveness in identifying non-compliant products. Finally, to capture the underlying food-safety conditions against which regulatory efforts operate, this study further introduces the random inspection failure rate as a proxy for regulatory outcomes. Compared with supervised inspections, random inspections provide a less biased assessment of overall food-safety conditions, as they are less influenced by regulatory targeting.

### 2.3 Food-safety social harm

Consumers worldwide face food-safety challenges to varying extents (22). Failures may occur at any point along the farm-to-table continuum. As a result, food-safety incidents are largely stochastic with respect to type, location, production stage, and responsible actor, and do not systematically privilege any single dimension. Accordingly, employing the number of food-safety incidents and the incidence of foodborne illness as indicators of regional food-safety performance is both reasonable and defensible (19). To ensure comparability across regions with different economic scales, the number of food-safety incidents is normalized by GDP (16). This normalization is intended to capture incident intensity relative to regional economic activity, thereby reducing scale bias and allowing for cross-regional comparability in food-safety risk exposure. It should also be noted that both food-safety incident data and foodborne illness statistics may be subject to reporting bias, as media coverage and official reporting practices can vary across regions and over time. In particular, media-reported incidents may overrepresent high-visibility events while underreporting less severe or less publicized cases. Therefore, the constructed indicators should be interpreted as observed social harm under reporting and monitoring constraints, rather than the exact underlying incidence of food-safety failures.

## 3 Methods and data sources

### 3.1 Methods

#### 3.1.1 Entropy-weighted TOPSIS method

The entropy-weighting method is a mathematical approach used to assess the degree of dispersion in indicator data. As dispersion increases, an indicator exerts a more pronounced influence on the overall evaluation, and its assigned weight correspondingly rises. As an objective quantitative weighting scheme, the entropy method determines indicator weights based on the information content observed in the data, thereby mitigating subjective bias arising from human judgment (23). The corresponding mathematical formulations of the entropy-weighting method are presented in Equations 1–9.

The mathematical formulation is as follows:

(1) Standardization of evaluation indicators

The range method (min–max normalization) is used to standardize the evaluation indicators:


$$\begin{array}{l} Y_{ij}=\{\begin{array}{c} \frac{X_{ij-}\min\left(X_{ij}\right)}{max(X_{ij)}-\min\left(X_{ij}\right)},if\;X_{ij}\;is\;a\;positive\;indicator\\ \frac{\max\left(X_{ij}\right)-X_{ij}}{max(X_{ij)}-\min\left(X_{ij}\right)},if\;X_{ij}\;is\;a\;negative\;indicator \end{array} \end{array}$$ (1)

where *i* and *j* denote the province and evaluation indicator, respectively; *X*_*ij*_ and *Y*_*ij*_ represent the original and standardized values of the evaluation indicator; and *max* (*X*_*ij*_) and min(*X*_*ij*_) denote the maximum and minimum values of *X*_*ij*_, respectively.

All indicators are first classified into positive and negative types according to their theoretical relationship with food safety. Specifically, positive indicators indicate that higher values correspond to better food safety performance, whereas negative indicators indicate that higher values correspond to poorer food safety performance. Through the normalization process, all negative indicators are transformed so that larger standardized values consistently represent better food safety performance. Therefore, after standardization, all indicators are directionally aligned, ensuring comparability across dimensions.

(2) Calculation of information entropy

The information entropy of each standardized indicator is calculated as:


$$\begin{array}{l} E_{j}=\ln\frac{1}{n}\sum_{i=1}^{n}[(Y_{ij}/\sum_{i=1}^{n}Y_{ij})]\ln(Y_{ij}/\sum_{i=1}^{n}Y_{ij})] \end{array}$$ (2)

(3) Calculation of indicator weights

The weight of each indicator is determined as follows:


$$\begin{array}{l} W_{j}=\frac{1-E_{j}}{\overset{m}{\underset{j=1}{\sum }}\left(1-E_{j}\right)} \end{array}$$ (3)

(4) Construction of the weighted decision matrix

The weighted matrix is constructed as:


$$\begin{array}{l} R={\left(r_{ij}\right)}_{n\times m} \end{array}$$ (4)

Where, *r*_*ij*_ = *W*_*j*_×*Y*_*ij*_

After weighting, all indicators enter the decision matrix in a unified direction, where larger values indicate better food safety performance.

(5) Determination of the positive and negative ideal solutions

Based on the weighted matrix *R*, the positive ideal solution and negative ideal solution are defined as:


$$\begin{array}{l} Q_{j}^{+}=\left(maxr_{i1},maxr_{i2},\ldots ,maxr_{im},\right) \end{array}$$ (5)

$$\begin{array}{l} Q_{j}^{-}=\left(minr_{i1},minr_{i2},\ldots ,minr_{im},\right) \end{array}$$ (6)

In this step, the positive ideal solution represents the best observed food safety performance across all indicators, while the negative ideal solution represents the worst observed performance. Since all indicators have been directionally standardized, the positive ideal solution consistently reflects “higher food safety level” across all dimensions.

(6) Calculation of Euclidean distances

The Euclidean distances between each evaluation object and the positive and negative ideal solutions are calculated as:


$$\begin{array}{l} d_{i}^{+}=\sqrt{\overset{m}{\underset{j=1}{\sum }}{\left(Q_{j}^{+}-r_{ij}\right)}^{2}} \end{array}$$ (7)

$$\begin{array}{l} d_{i}^{-}=\sqrt{\overset{m}{\underset{j=1}{\sum }}{\left(Q_{j}^{-}-r_{ij}\right)}^{2}} \end{array}$$ (8)

(7) Calculation of relative closeness

The relative closeness to the ideal solution is calculated as:


$$\begin{array}{l} C_{i}=\frac{d_{i}^{-}}{d_{i}^{+}+d_{i}^{-}} \end{array}$$ (9)

The resulting relative closeness coefficient *C*_*i*_ is thus a unified composite index of food safety performance, where a higher value of *C*_*i*_ indicates a higher overall level of food safety.

#### 3.1.2 Dagum Gini decomposition method

The Dagum Gini coefficient and its decomposition were originally proposed to quantify income inequality and have since been widely applied to the analysis of regional development disparities (24). A key limitation of the conventional Gini coefficient and the Theil index is that they can overestimate the contributions of within-region and between-region disparities to overall inequality. The mathematical expressions of the Dagum Gini decomposition method are provided in Equations 10–20.

The overall Gini coefficient is defined as:


$$\begin{array}{l} G=\sum_{j=1}^{k}\sum_{h=1}^{k}\sum_{i=1}^{k}\sum_{r=1}^{k}\frac{\left|y_{ji}-y_{hr}\right|}{2n^{2}\bar{y}} \end{array}$$ (10)

where *G* denotes the overall Gini coefficient; *k* and *n* represent the number of regions and provinces, respectively; *n*_*j*_ and *n*_*h*_ denote the numbers of provinces in regions *j* and *h*; *y*_*ji*_ and *y*_*hr*_ represent the food-safety performance of province *i* in region *j* and province *r* in region *h*, respectively; and $\overset{-}{y}$ denotes the overall mean.

The intra-regional Gini coefficient for region *j* is calculated as:


$$\begin{array}{l} G_{jj}=\frac{\sum_{i=1}^{nj}\sum_{r=1}^{nj}\left|y_{ji}-y_{hr}\right|}{2n^{2}\bar{y}} \end{array}$$ (11)

The inter-regional Gini coefficient between regions *j* and *h* is defined as:


$$\begin{array}{l} G_{jh}=\frac{\sum_{i=1}^{nj}\sum_{r=1}^{nh}\left|y_{ji}-y_{hr}\right|}{n_{j}n_{h}({\bar{y}}_{j}+{\bar{y}}_{h})} \end{array}$$ (12)

where ${\overset{-}{y}}_{j}$ and ${\overset{-}{y}}_{h}$ denote the mean food-safety performance in regions *j* and *h*, respectively. *G*_*jj*_ represents intra-regional inequality, while *G*_*jh*_ captures inter-regional inequality between regions *j* and *h*.

The overall Gini coefficient can be decomposed into three components: *G* = *GW*+*Gnb* +*Gt*.

Where *G*_*w*_ represents intra-regional disparity, *G*_*nb*_ represents inter-regional disparity, *G*_*t*_ represents the transvariation density (overlapping component).

The intra-regional disparity component is calculated as:


$$\begin{array}{l} G_{w}=\overset{k}{\underset{j=1}{\sum }}G_{jj}p_{j}s_{j} \end{array}$$ (13)

The inter-regional disparity component is defined as:


$$\begin{array}{l} G_{nb}=\overset{4}{\underset{j=2}{\sum }}\overset{j-1}{\underset{h-1}{\sum }}G_{jh}\left(p_{j}s_{h}+p_{h}s_{j}\right)D_{jh} \end{array}$$ (14)

The transvariation density component is calculated as:


$$\begin{array}{l} G_{t}=\sum_{j=2}^{4}\sum_{h-1}^{j-1}G_{jh}(p_{j}s_{h}+p_{h}s_{j})(1-D_{jh}) \end{array}$$ (15)

In Equations 13–15:


$$\begin{array}{l} p_{j}=\frac{nj}{n} \end{array}$$ (16)

$$\begin{array}{l} s_{j}=\frac{\left(\;nj\;\bar{y}j\right)}{n\bar{y}} \end{array}$$ (17)

where *p*_*j*_ represents the population share of region *j*, and *s*_*j*_ represents the income share (or food-safety performance share) of region *j*.

The relative economic affluence between regions is captured by the parameter *D*_*jh*_, defined as:


$$\begin{array}{l} D_{jh}=\frac{d_{jh}-p_{jh}}{d_{jh}+p_{jh}} \end{array}$$ (18)

where


$$\begin{array}{l} d_{jh}=\overset{\infty }{\underset{0}{\int }}dF_{j}\left(y\right)\overset{y}{\underset{0}{\int }}\left(y-x\right)dF_{h}\left(x\right) \end{array}$$ (19)

$$\begin{array}{l} d_{jh}=\overset{\infty }{\underset{0}{\int }}dF_{h}\left(y\right)\overset{y}{\underset{0}{\int }}\left(y-x\right)dF_{h}\left(x\right) \end{array}$$ (20)

where *F*_*j*_(*y*)and *F*_*h*_(*y*)denote the cumulative distribution functions of food-safety performance in regions *j* and *h*, respectively. The terms *d*_*jh*_ and *p*_*jh*_ represent the inter-regional difference intensity and the transvariation intensity, respectively.

#### 3.1.3 Kernel density estimation (KDE)

Kernel density estimation (KDE) introduces a kernel function for each sample value and convolves these kernels to obtain the final density estimate. As an effective non-parametric tool, KDE quantifies spatial heterogeneity by depicting a smooth, continuous density curve, thereby revealing the distributional characteristics of a random variable (25). This approach substantially reduces the subjectivity associated with specifying parametric functional forms. Owing to these advantages, KDE has been widely employed in studies of spatial imbalance. In this paper, KDE is used to examine the location, shape, and dispersion of the distribution of China's food-safety performance index. The KDE procedure is specified as follows:


$$\begin{array}{l} f\left(x\right)=\frac{1}{N_{h}}\overset{N}{\underset{i=1}{\sum }}K\left(\frac{X_{i}-x}{h}\right) \end{array}$$ (21)

In Equation 21, *K*(·) denotes the kernel function, *N* and *h* represent the number of observations and the bandwidth, respectively, and *x* denotes the mean value. The Gaussian kernel function is employed to characterize the dynamic evolution of food-safety performance in China, as specified in Equation 22.


$$\begin{array}{l} f\left(x\right)=\frac{1}{\sqrt{2\pi }}exp\left(-\frac{x^{2}}{2}\right) \end{array}$$ (22)

### 3.2 Data Sources

The data used in this study were obtained from two main sources. First, we assembled web-based data, including food-safety supervisory sampling inspection records disclosed on the official websites of provincial and municipal market supervision administrations (approximately 3.91 million inspection batches), information on HACCP-certified enterprises from the National Public Service Platform for Certification and Accreditation Information, and province-level counts of food enterprises retrieved from the China QS inquiry website. The panel dataset covers 30 provincial-level administrative regions in China during 2016–2021, including regions with substantial differences in economic development, industrial structure, population size, and regulatory capacity. Therefore, the sample reflects considerable economic and social heterogeneity across China. Tibet was excluded because of continuous missing observations for several key variables during the study period, which prevented the construction of a balanced panel dataset. Second, we drew statistical indicators from a set of authoritative yearbooks, namely the *China Statistical Yearbook*, the *China Food Industry Statistical Yearbook*, the *China Information Industry Statistical Yearbook*, the *China Industrial Statistical Yearbook*, as well as statistical yearbooks published by individual provinces and municipalities. Because the *China Industrial Statistical Yearbook* lacks data for 2017 and 2018, missing observations for some provinces were supplemented using the *Statistical Communiqués on National Economic and Social Development* available through the government information disclosure portals of the relevant official websites. It should be noted that the study period (2016–2021) overlaps with the COVID-19 pandemic. This may have affected certain administrative processes, regulatory enforcement intensity, and the reporting of food-safety incidents. However, as the data are drawn from official administrative records and statistical yearbooks, and the study focuses on structural evaluation rather than short-term fluctuations, the potential influence of the pandemic is expected to be limited in affecting the overall measurement results.

## 4 Evaluation results

### 4.1 Dimension-specific evaluation

Table 2 presents the dimension-specific and composite evaluation results of food safety for each province in China. As the core actors responsible for food production and operation, producers' compliance and managerial practices are closely linked to the baseline quality of food-safety performance. Accordingly, this dimension is assessed using key indicators such as producers' certification practices and non-compliant behaviors. Prior research indicates that producers' food-safety performance is strongly associated with regional socioeconomic development and the completeness of local industrial supporting systems. In economically advanced regions, producers are more likely to adopt modern production technologies and preventive quality-management systems (e.g., HACCP), which may enhance their capacity and incentives to improve food safety (26). The 2021 results exhibit pronounced spatial differentiation. Beijing (0.981) ranks first nationwide. As China's political, economic, and cultural center, it concentrates leading food enterprises that are at the forefront domestically in deploying intelligent manufacturing equipment and establishing end-to-end quality traceability systems. Shanghai (0.566) and Tianjin (0.380) follow, and the three municipalities benefit from well-developed industrial ecosystems, rapid technological upgrading, and stringent self-regulation among producers—advantages that are associated with stronger performance in raw-material control and process standardization. In contrast, lower-ranked provinces such as Gansu (0.026), Heilongjiang (0.031), and Shaanxi (0.051) are characterized by relatively weak industrial foundations. Financial constraints among small and micro enterprises may limit investments in technological upgrading and quality management, which may be associated with structural deficiencies in producer-side capacity and are therefore associated with weaker food-safety performance (27).

**Table 2:** Composite food safety index and dimension scores across Chinese provinces in 2021.

Province	Food-safety behavior of producers	Government food-safety regulation	Food safety-social harm	Composite food-safety index
Beijing	0.981	0.878	0.934	0.898
Tanjin	0.380	0.102	0.988	0.213
Hebei	0.301	0.072	0.973	0.169
Shanxi	0.066	0.096	0.917	0.094
Neimenggu	0.075	0.100	0.813	0.098
Liaoning	0.077	0.071	0.994	0.081
Jilin	0.068	0.202	0.870	0.176
Heilongjiang	0.031	0.205	0.973	0.175
Shanghai	0.566	0.208	0.972	0.335
Jiangsu	0.142	0.128	0.920	0.136
Zhejiang	0.143	0.093	0.844	0.113
Anhui	0.099	0.076	0.946	0.090
Fujian	0.173	0.124	0.833	0.142
Jiangxi	0.095	0.076	0.897	0.088
Shandong	0.107	0.121	0.695	0.120
Henan	0.177	0.130	0.953	0.149
Hubei	0.106	0.070	0.964	0.088
Hunan	0.083	0.075	0.827	0.083
Guangdong	0.279	0.080	0.947	0.162
Guangxi	0.052	0.053	0.901	0.061
Hainan	0.055	0.241	0.721	0.203
Chongqing	0.095	0.540	0.895	0.445
Sichuan	0.214	0.069	0.876	0.131
Guizhou	0.115	0.272	0.710	0.237
Yunnan	0.143	0.165	0.361	0.159
Shaaxi	0.051	0.097	0.872	0.091
Gansu	0.026	0.073	0.871	0.069
Qinghai	0.272	0.399	0.907	0.364
Ningxia	0.085	0.276	0.863	0.236
Xinjiang	0.127	0.320	0.976	0.273

Government regulation constitutes the central pillar of food-safety governance, and its performance is closely related to both the implementation capacity and the effective coverage of food-safety initiatives. This dimension is therefore measured using core indicators including inspection pass rates, regulatory intensity, and regulatory efficiency. Regulatory oversight is generally associated with producers' food-safety behavior: greater probabilities of detection and more stringent penalties are often linked to higher compliance, whereas high monitoring costs that hinder the identification of violations and weak sanctions that reduce deterrence may be associated with lower regulatory effectiveness and slower improvements in food-safety performance (7). The empirical results show marked regional heterogeneity. In 2021, the top three performers on this dimension are Beijing (0.878), Chongqing (0.540), and Qinghai (0.399). Beijing leverages its administrative and enforcement capacity to achieve strong performance in standardized law enforcement and broad regulatory coverage. Chongqing and Qinghai, by contrast, also exhibit relatively strong regulatory performance, which may be related to increasing local oversight investments and improving cross-departmental coordination mechanisms, which is associated with more favorable regulatory outcomes. At the lower end of the distribution, Guangxi Zhuang Autonomous Region (0.053), Sichuan (0.069), and Hubei (0.070) rank last. These provinces are dominated by mountainous and hilly terrain, which raises supervisory costs and may contribute to regulatory blind spots in remote areas, and is associated with weaker regulatory performance. Collectively, these differences underscore the role of government regulation as a “visible hand” in correcting market failures and are consistent with its potential importance in promoting food-safety performance (28).

The social-harm dimension of food safety captures the overall consequences of food-safety performance for public health, social stability, and economic performance. Its assessment is closely tied to the prevention and control of biological, physical, and chemical hazards across the entire food supply chain (13, 14) and is operationalized through key indicators, including the number of food-safety incidents and the number of foodborne disease cases. The results show that Liaoning (0.994), Tianjin (0.987), and the Xinjiang Uygur Autonomous Region (0.976) rank among the top three regions on this dimension, whereas Yunnan (0.361), Shandong (0.695), and Guizhou (0.709) occupy the bottom positions. Variation in social-harm scores reflects differences in regional food-safety risk exposure and is associated with factors such as climate change, regulatory capacity, and levels of economic development (27).

### 4.2 Comprehensive evaluation

Based on the entropy-weighted TOPSIS approach, we computed weighted scores for the three dimensions—producers' food-safety behavior, government regulatory oversight, and the social-harm consequences of food safety—and thereby constructed a composite food-safety evaluation index for 30 Chinese provinces and municipalities over the period 2016–2021. The 2021 results show that Beijing (0.898), Chongqing (0.444), and Qinghai (0.364) rank among the top performers, with Beijing occupying the leading position and exhibiting a pronounced advantage over other regions. At the lower end of the distribution, Liaoning (0.080), Gansu (0.069), and Guangxi (0.061) rank last; these provinces are generally characterized by relatively low levels of economic development. From a spatial perspective, the regional pattern displays a clear gradient—Eastern > Western > Northeastern > Central—as reported in Table 3.

**Table 3:** Composite food safety index and dimension scores across four major regions of China in 2021.

Region	Food-safety behavior of producers	Government food-safety regulation	Food-safety social harm	Composite food-safety index
Eastern	0.313	0.205	0.883	0.249
Central	0.104	0.087	0.917	0.098
Western	0.114	0.215	0.822	0.196
Northeastern	0.059	0.160	0.946	0.144

The Eastern region exhibits the highest overall food-safety performance among the four macro-regions (0.249). Dimension-specific results show that it leads in producers' food-safety behavior (0.313) and maintains a comparatively strong performance in government regulation (0.205). By contrast, the East does not display a commensurate advantage in the social-harm dimension (0.883), implying that further gains may depend increasingly on reducing the downstream public-health and societal consequences of food-safety risks. This pattern is consistent with the East's structural advantages. Its stronger economic base is associated with lower barriers to the adoption of advanced food-processing equipment and may facilitate the diffusion of HACCP (Hazard Analysis and Critical Control Points) certification, thereby being linked to improved quality assurance at the production source. Moreover, the region's broader and deeper application of digital technologies, together with more extensive online–offline integration in the food sector, may enhance the contribution of the digital economy to traceability, monitoring, and risk-management capabilities. Finally, sustained economic strength supports the attraction of regulatory talent and continual upgrading of technical infrastructure, further consolidating regulatory capacity (29).

The Western region ranks second among the four macro-regions in overall food-safety performance (0.196). The dimension-level results show that it places second in producers' food-safety behavior (0.114) and leads all regions in government regulation (0.215), while its social-harm score remains comparatively low (0.822). Collectively, these indicators point to a broadly positive trajectory of food-safety development in the West. In particular, sustained efforts to strengthen producer-side management and regulatory oversight appear to have contributed to continued improvements in regional food-safety conditions. This pattern is broadly consistent with a Kuznets-type relationship between development and food safety suggested in prior studies (13, 14). As the West's industrialization process approaches a developmental turning point, industrial upgrading can enhance the institutional, technological, and infrastructural foundations of food-safety governance, thereby supporting further improvements in food-safety outcomes (30).

The Northeastern region ranks third among the four macro-regions in overall food-safety performance (0.144). At the dimension level, it records the lowest score for producers' food-safety behavior (0.059) and a relatively high score on the social-harm dimension (0.946), while the enabling contribution of government regulation is comparatively modest (0.160). Together, these results help explain the Northeast's weaker overall food-safety performance. This pattern may reflect the region's industrial and fiscal constraints. Although the Northeast has a long-established food-processing sector, its comparatively sluggish economic growth in recent years may have slowed the renewal of production equipment and the adoption of advanced technologies relative to other regions. Moreover, effective food-safety regulation requires stable financial support, and local fiscal capacity is largely shaped by the broader level of economic development. The Northeast's relative economic underperformance may therefore limit regulatory funding and enforcement capability, thereby constraining governance effectiveness (13, 14).

The Central region records the lowest overall food-safety performance among the four macro-regions (0.098), effectively constituting a “depressed area” of food-safety development. This characterization is broadly consistent with China's spatial gradient in economic development—East > Central > West—and with prior evidence that economic development is closely intertwined with food safety (13, 14, 31). The dimension-level results further clarify the sources of this disadvantage: the Central region ranks third in producers' food-safety behavior (0.104), last in government regulation (0.087), and second in the social-harm dimension (0.917). Several mechanisms may underlie this pattern. First, relatively weak economic growth may be associated with reduced regulatory effectiveness through constraints on fiscal resources for oversight, which may in turn limit the region's capacity to attract and retain skilled regulators and be associated with lower levels of investment in technical facilities and infrastructure upgrades (32). Second, as both a major grain-producing area and a high population-density region, the Central region faces particularly complex food circulation networks, which may be associated with higher monitoring costs and exacerbate enforcement challenges. These constraints may be reflected in comparatively low sampling inspection pass rates and inspection efficiency, contributing to the region's weak performance on the government regulation dimension. Finally, the Central region does not exhibit a clear advantage on the social-harm dimension relative to other regions; combined with regulatory underperformance, this may be associated with its composite food-safety index remaining persistently low.

Following Wei and Li (33), we classify the 2021 food-safety performance of the 30 provinces and municipalities based on the sample mean and standard deviation of the composite index. Let M denote the mean and SD denote the standard deviation. Provinces with index values exceeding M + 0.5SD are classified as leading, those with values within the interval (M – 0.5SD, M + 0.5SD) are classified as catching-up, and those with values below M – 0.5SD are classified as lagging.

As shown in Table 4, provinces in the Eastern region are concentrated in the leading and catching-up categories, whereas most provinces in the Central, Western, and Northeastern regions fall into the catching-up and lagging groups. Notably, the Western region exhibits a more balanced distribution across all three categories, indicating substantial intra-regional heterogeneity and a wide range of food-safety performance among Western provinces.

**Table 4:** Regional distribution of food-safety performance types in China, 2021.

Region	Eastern region	Central region	Western region	Northeastern region
Leading type	Beijing, Shanghai		Chongqing, Qinghai, Xinjiang	
Catching-up type	Tianjin, Hebei, Jiangsu, Zhejiang, Fujian, Shandong, Guangdong, Hainan	Henan	Sichuan, Guizhou, Yunnan, Ningxia	Jilin, Heilongjiang
Lagging type		Shanxi, Anhui, Jiangxi, Hubei, Hunan	Inner Mongolia, Guangxi, Shaanxi, Gansu	

## 5 Assessment of regional disparities and dynamic evolution

### 5.1 Temporal evolution and structural features

**Figure 1:**
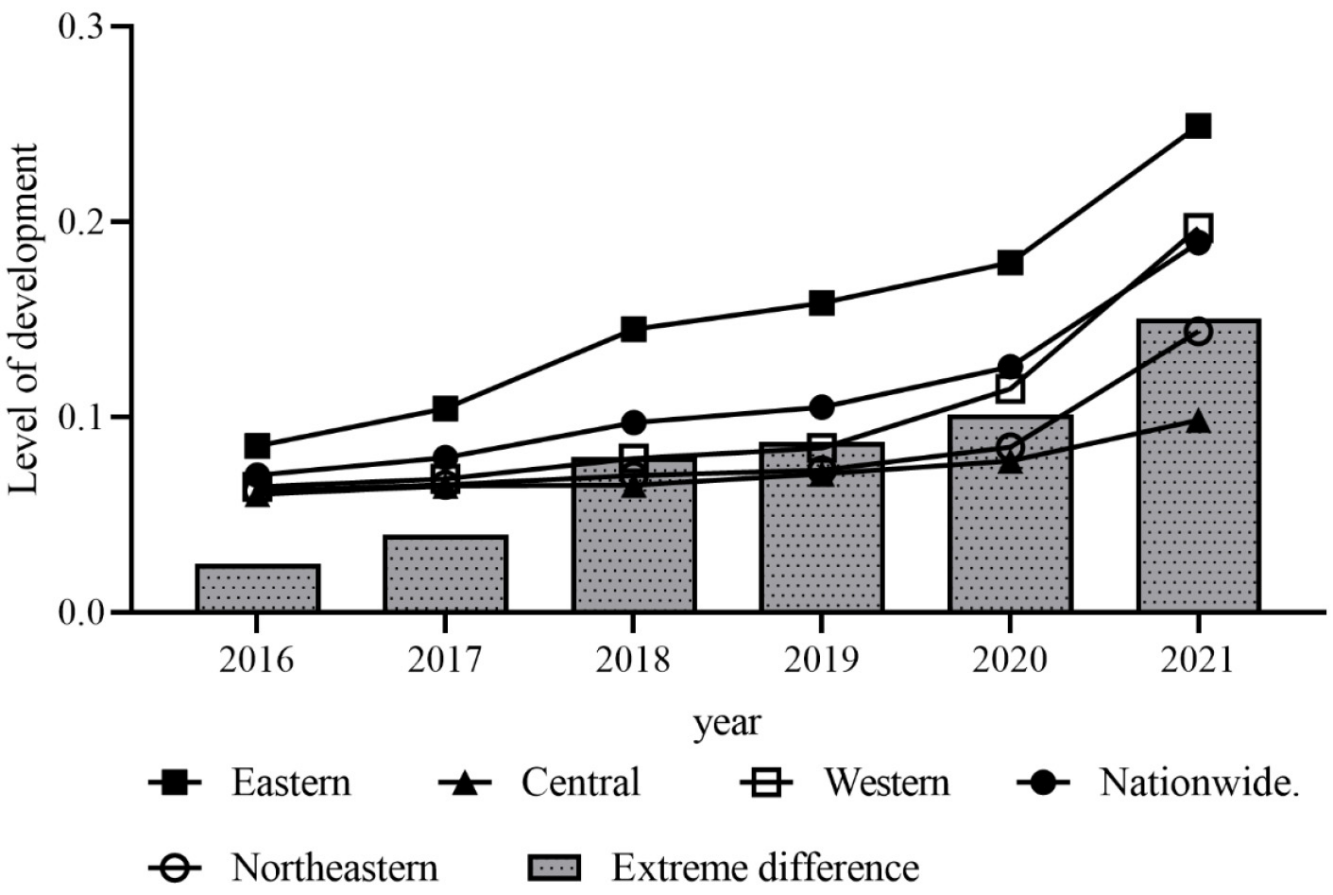
Temporal evolution and structural characteristics of food safety performance in China.

Figure 1 depicts the time-series evolution and structural features of China's food-safety performance. Two main patterns emerge. (1) At the national level, the food-safety performance increases steadily over time, rising from 0.070 in 2016 to 0.189 in 2021, representing an overall increase of 169%. This improvement likely reflects the policy impetus of the “Four Strictest” food-safety requirements introduced in 2015, under which national food-safety conditions have progressively strengthened. (2) At the regional level, all four macro-regions exhibit upward trajectories, although the pace of improvement differs markedly. The Eastern region rises from 0.085 to 0.249 (193%), the Central region from 0.060 to 0.099 (65%), the Western region from 0.064 to 0.197 (207%), and the Northeastern region from 0.062 to 0.144 (132%), with the ordering of growth rates being Western > Eastern > Northeastern > Central. The spatial dynamics further indicate divergence. The Eastern region maintains rapid growth throughout the period, consistently widening its advantage over other regions. Although the Western region exhibited a small initial gap in 2016, it subsequently experienced accelerated improvement—likely supported by the “Four Strictest” policy framework and interregional assistance mechanisms—thereby overtaking both the Central and Northeastern regions. In contrast, the Central and Northeastern regions grow more slowly, reflecting weaker endogenous development momentum; the Central region, in particular, falls further behind. Notably, the Northeastern region has accelerated in recent years, surpassing the Central region.

### 5.2 Assessment of regional disparities and their sources

This study applies the Dagum Gini coefficient to investigate regional disparities in food-safety performance across China. The empirical results are presented in Table 5.

**Table 5:** Regional disparities in food safety performance in China and their decomposition.

Year	Gini coefficient	Intra-regional disparity			Inter-regional disparity			Contribution rate (%)		
		Eastern	Central	Western	East-Central	East-West	Central-West	Intra-regional	Inter-regional	Transvariation density
2016	0.193	0.224	0.076	0.191	0.188	0.220	0.191	33.019	36.806	30.175
2017	0.235	0.258	0.087	0.217	0.232	0.263	0.217	31.700	45.460	22.839
2018	0.294	0.237	0.076	0.211	0.316	0.317	0.211	30.373	57.180	12.447
2019	0.300	0.345	0.082	0.203	0.328	0.326	0.203	30.669	56.035	13.296
2020	0.315	0.358	0.086	0.246	0.335	0.329	0.246	31.432	49.039	19.529
2021	0.362	0.383	0.169	0.338	0.358	0.372	0.338	32.649	35.078	32.273
Mean	0.283	0.301	0.096	0.234	0.293	0.305	0.234	31.640	46.600	21.760

#### 5.2.1 Overall disparity

**Figure 2:**
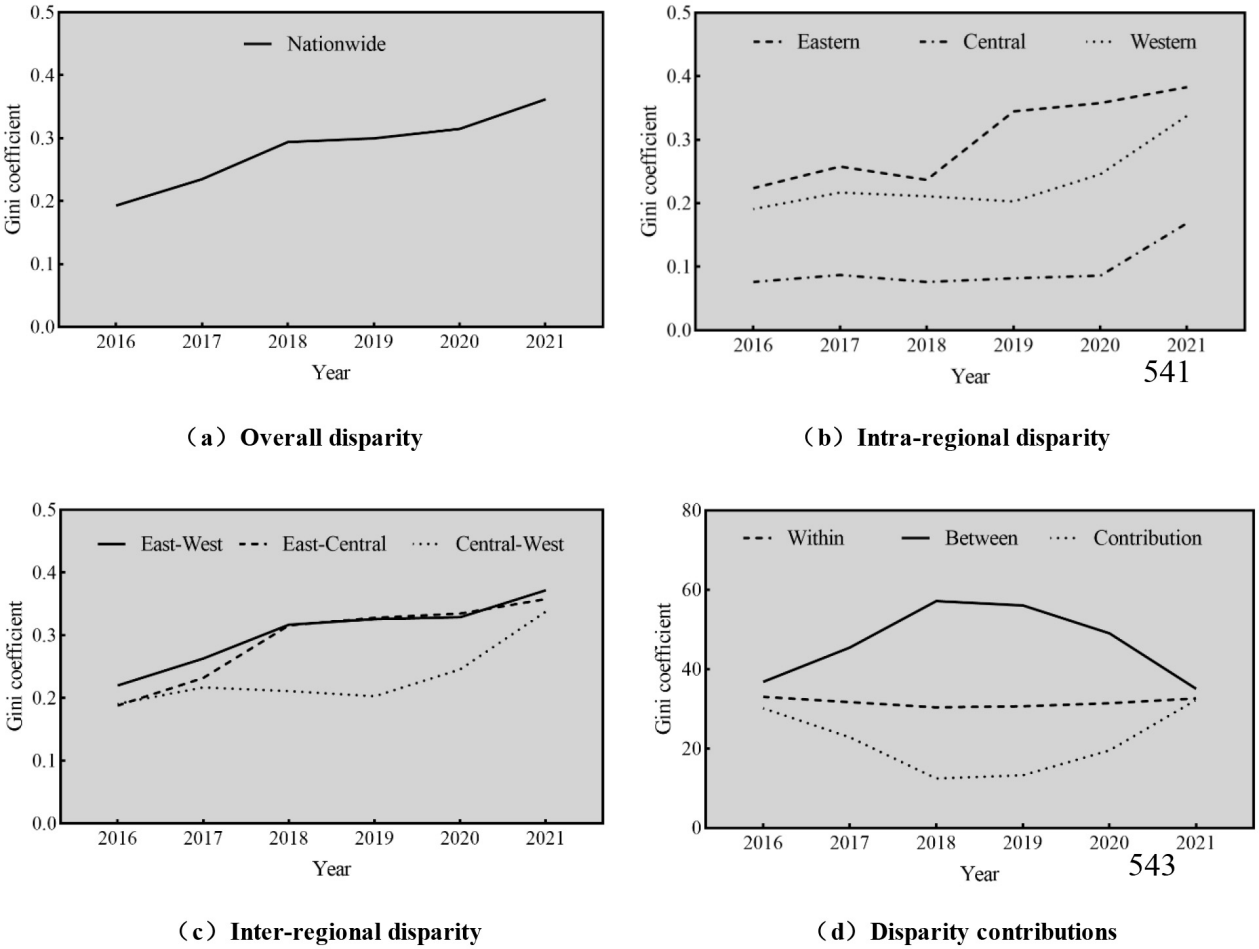
Regional disparities and their sources in China's food-safety performance. **(a)** Overall disparity. **(b)** Intra-regional disparity. **(c)** Inter-regional disparity. **(d)** Disparity contributions.

As illustrated in Figure 2a, the Gini coefficient of China's food-safety performance exhibits a clear upward trajectory over 2016–2021. Specifically, the overall Gini rises from 0.193 in 2016 to 0.362 in 2021—an increase of 88%—indicating that regional disparities in food-safety performance have widened steadily. This divergence is plausibly linked to structural differences in economic development and urbanization. Over the past decade, the Eastern region has generally advanced more rapidly in both economic growth and urbanization, whereas the Central and Western regions, despite policy support, remain substantially behind. Such uneven development, compounded by marked heterogeneity across provinces, is mirrored in increasingly pronounced interprovincial gaps in food-safety performance.

#### 5.2.2 Sources of overall disparities

As shown in Figure 2d, the source structure of regional disparities in China's food-safety performance exhibits a “balanced–imbalanced–balanced” evolutionary pattern, with inter-regional disparity remaining the dominant contributor overall. More specifically, the contribution of inter-regional disparity exhibits an inverted U-shaped trajectory over time, whereas the contributions of intra-regional disparity and transvariation density follow U-shaped trends. The mean contribution rates are 46.600%, 31.640%, and 21.760% for inter-regional disparity, intra-regional disparity, and transvariation density, respectively. Inter-regional disparity accounted for 36.806% in 2016, peaked at 57.180% in 2018, and declined to 35.078% by 2021. In contrast, intra-regional disparity changed relatively smoothly over time, reaching its minimum in 2018.

#### 5.2.3 Decomposition of disparity sources

To further investigate the sources of regional disparities in China's food-safety performance, the study decomposes the differences into intra-regional and inter-regional disparities.

Intra-regional disparities in food safety performance have widened substantially in the Eastern, Central, and Western regions compared with 2016 (Figure 2b). The Eastern region exhibits an overall upward trend with short-term fluctuations, although the rate of divergence has moderated in recent years. This moderation may reflect progress under China's “common prosperity” agenda, with several Eastern provinces piloting reforms aimed at reducing internal inequality (34). As a key component of basic livelihood protection, food safety has consequently become an important policy priority. The Western region also shows an overall upward but volatile trend, entering a phase of more pronounced divergence after 2019. Although the Central region maintains the lowest intra-regional disparity among the three regions, its internal disparity has also increased notably since 2020. These findings suggest that future policy should place greater emphasis on reducing intra-regional inequality, particularly by promoting more balanced food safety performance in the Central and Western regions.

Inter-regional disparities exhibit heterogeneous dynamic patterns (Figure 2c). The East–West and East–Central disparities both increase steadily over time and remain broadly comparable in magnitude. In contrast, the Central–West disparity follows a U-shaped trajectory. Notably, the Central–West disparity widen markedly after 2019 and gradually converges toward the levels observed in the East–West and East–Central comparisons. These findings suggest that greater policy attention should be directed toward improving food safety performance in the Central and Western regions, in order to prevent further acceleration of inter-regional divergence that could exacerbate cross-regional spillover risks in food safety.

### 5.3 Assessment of dynamic evolution

**Figure 3:**
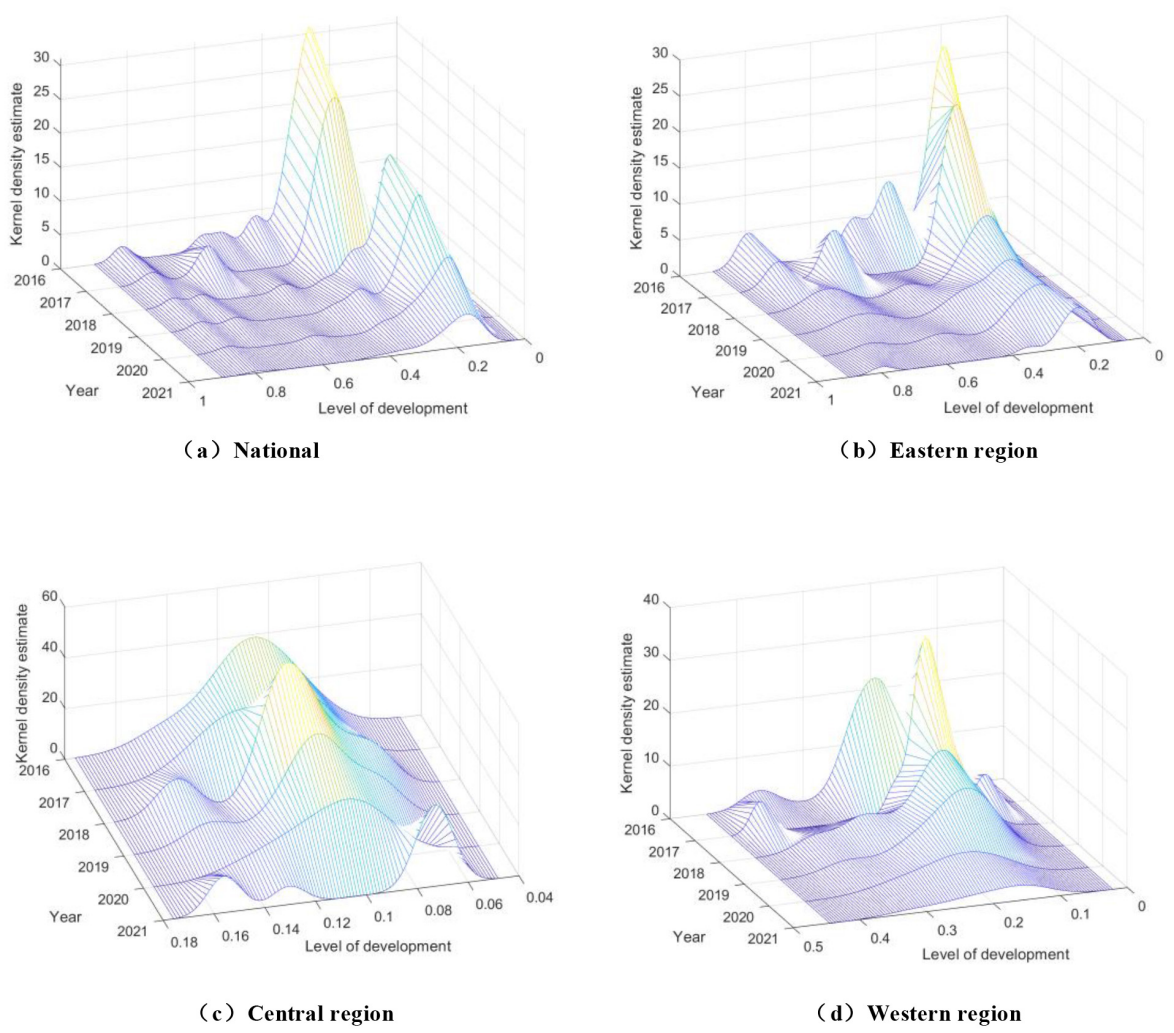
Dynamic evolution of food safety performance in China and the Eastern, Central, and Western regions. **(a)** National. **(b)** Eastern region. **(c)** Central region. **(d)** Western region.

This study employs kernel density estimation to characterize the temporal dynamics of China's food-safety performance and to analyze key distributional features—such as shifts in location and changes in shape. The results are presented in Figure 3.

As shown in Figure 3a, at the national level, the peak of the kernel density curve gradually declines over time. Following initial fluctuations, the distribution develops an increasingly pronounced left tail and evolves from a multimodal to a unimodal pattern. This evolution indicates a general upward shift in China's food-safety performance. Meanwhile, the reduced peak height and unimodal transition suggest diminishing polarization and greater concentration around the central tendency. However, the simultaneous expansion of the distributional range indicates that regional disparities have continued to widen, with the gap between the highest and lowest levels increasing over time.

At the regional level, the kernel density distribution for the Eastern region shifts leftward, with peak height steadily declining, peak width gradually increasing, and the number of peaks decreasing markedly (Figure 3b). These patterns indicate a continuous improvement in the food-safety performance in the Eastern region, accompanied by reduced dispersion and relatively weak polarization. However, regional disparities have widened, as reflected in an increasing distributional range (i.e., a larger max–min gap). For the Central region, the kernel density curve also shifts leftward overall, with peak height gradually decreasing and peak width narrowing, while the number of peaks evolves from unimodal to multimodal (Figure 3c). This suggests that the food-safety performance in the Central region has steadily improved, but dispersion has increased and polarization has intensified. At the same time, the overall range has narrowed, indicating a reduction in the extreme gap between the highest and lowest values. In the Western region, the kernel density curve exhibits fluctuations in the early period and shifts leftward in the later period. Peak height fluctuates initially but declines over time, peak width gradually increases, and the number of peaks steadily decreases (Figure 3d). These changes indicate that the food-safety performance in the Western region experienced early volatility but has risen steadily in recent years. Meanwhile, regional disparities have expanded, as reflected in a widening distributional range, although polarization has gradually weakened.

## 6 Conclusions

Drawing on a three-dimensional framework that integrates producers, government, and consumers, this study provides a comprehensive assessment of food-safety performance across 30 Chinese provinces and municipalities over the period 2016–2021. We further employ Dagum Gini decomposition to disentangle the sources of regional disparities and apply kernel density estimation to characterize the distributional dynamics over time. On this basis, we arrive at the following key conclusions:

First, China's food-safety performance has continued to improve over time. The current spatial pattern can be summarized as “Eastern > Western > Northeastern > Central.” Notably, the Central region emerges as a pronounced lagging area in food-safety performance; therefore, closing this gap and accelerating improvement in the Central region is likely to be a key lever for raising China's overall food-safety performance.

Second, regional disparities in China's food-safety performance have widened progressively over time. The decomposition results indicate that inter-regional disparity remains the dominant contributor to overall inequality, although its contribution has gradually declined. Within the inter-regional component, the East–Central and Central–West disparities account for the largest shares, with the Central–West disparity, in particular, having expanded markedly in recent years. In contrast, intra-regional disparity remains relatively stable overall. The Eastern and Western regions contribute most to intra-regional inequality, whereas the Central region has experienced a significant increase in internal disparity, reflecting a growing internal imbalance in food-safety performance.

Third, the kernel density estimates suggest that food-safety performance has improved nationally and across the Eastern, Central, and Western regions, yet regional disparities have continued to widen. In the Eastern region, the distributional profile transitions from multimodality to a more clearly unimodal shape, indicating a gradual weakening of polarization. In the Central region, the density curve displays leftward fluctuations and an emerging multi-peaked structure, suggesting increasing multipolar differentiation. In the Western region, the distribution shifts leftward more prominently, consistent with a comparatively rapid improvement in food-safety performance.

These findings contribute to a more precise understanding of regional heterogeneity in China's food safety performance and provide important policy implications for the design of evidence-based and regionally differentiated governance strategies.

## 7 Policy recommendations

Building on the above findings and in light of regional development realities, we derive the following policy implications for narrowing regional disparities in food-safety performance and enhancing the overall effectiveness of food-safety governance nationwide:

First, address the Central region's structural weaknesses through a targeted development strategy. This requires increasing fiscal allocations for regulatory capacity, and improving institutional linkages between origin certification for agricultural products and downstream market-access controls. Priority should be given to supporting small and micro food producers in undertaking technological upgrading and expanding the adoption of HACCP certification. In parallel, an interregional regulatory coordination platform linking the Eastern and Central regions should be established. Given the complex circulation networks in major grain-producing provinces, coordinated oversight of critical nodes—particularly storage and transportation—should be strengthened to enhance end-to-end governance capacity along the food supply chain.

Second, optimize the spatial allocation of regulatory resources to promote more balanced regional development. This can be achieved by coordinating regulatory inputs across the Eastern, Central, and Western regions and institutionalizing mechanisms through which the Eastern region provides technical assistance and talent support to the Central, Western, and Northeastern regions. In particular, transferable best practices—such as digitalized sampling inspections and smart regulatory systems—should be disseminated and scaled. To curb the widening Central–Western gap, regulatory standards should be further harmonized across regions, and cross-jurisdictional policy coordination should be strengthened through instruments such as “regulatory alliances” and structured experience-sharing forums. Such measures can reduce governance fragmentation and mitigate uneven development arising from interregional differences in regulatory capacity and practice.

Third, accelerate the construction of a co-governance system for food safety. Building on a framework of “government leadership, producer accountability, societal participation, and technological empowerment,” food-safety education and risk communication should be delivered in a targeted manner across regions and population groups to strengthen consumers' supervisory awareness and participation capacity, particularly in the Central, Western, and Northeastern regions. Complaint and whistleblowing channels should be further streamlined, and incentive schemes for consumer reporting should be established. At the same time, the capacities of industry associations, the media, and research institutions should be integrated to foster a multi-actor governance architecture characterized by producer self-regulation, effective government oversight, and active public engagement.

## 8 Limitations

Despite the contributions of this study, several limitations should be acknowledged. First, potential multicollinearity and conceptual overlap among indicators may affect the robustness of the evaluation framework. Although the indicator system was designed to capture distinct dimensions of food safety—namely producers' behavior, government regulation, and social harm—certain variables (e.g., producer violation behavior and inspection failure rates) may reflect related aspects of compliance and regulatory detection. This overlap may introduce redundancy in the information captured and could influence the weights derived from the entropy method. Although entropy weighting relies on data variability rather than model-based estimation, and thus may partially mitigate the impact of multicollinearity, the possibility of biased weighting cannot be entirely ruled out. Future research could incorporate additional robustness checks, such as correlation analysis or dimensionality-reduction techniques (e.g., principal component analysis), to further assess the independence and validity of the selected indicators.

Second, the current evaluation framework does not explicitly account for certain important food-safety hazards, particularly those related to environmental contamination. For example, persistent organic pollutants (POPs) in food of animal origin—potentially arising from environmental exposure and individual consumption behaviors—represent a significant but underexplored source of risk. Existing evidence suggests that such contaminants may accumulate along the food chain and pose long-term health risks to consumers. The exclusion of these factors implies that the study's measurement of food-safety performance may not fully capture all dimensions of food-related health risks. Future research could extend the indicator system to incorporate environmental and behavioral risk factors, thereby providing a more comprehensive assessment of food safety.

## Data Availability

The original contributions presented in the study are included in the article/supplementary material, further inquiries can be directed to the corresponding author.

## Appendix

Drawing on the study by Li and Bo (35), this paper transforms the supervised inspection failure rate into a random inspection failure rate and uses the relationship between the two to measure the efficiency of food safety supervision inspections. The calculation is as follows:

$$\begin{array}{l} ratio\_efficiency=S/R \end{array}$$
(1)

$$\begin{array}{l} S=\left(\overset{T}{\underset{j=1}{\sum }}Q_{j}\right)÷\left(\overset{T}{\underset{j=1}{\sum }}N_{j}\right) \end{array}$$
(2)

$$\begin{array}{l} R=\overset{T}{\underset{j=1}{\sum }}V_{j}\times \left(M_{j}÷M\right) \end{array}$$
(3)

In Equations 1–3, *S* denotes the supervised inspection failure rate of a given province, and *R* represents the corresponding random inspection failure rate. *Q*_*j*_ is the number of failed batches identified in supervised inspections for food category *j*, and *N*_*j*_ is the total number of supervised inspection batches for that category. *V*_*j*_ denotes the supervised inspection failure rate for category *j*, *M*_*j*_ is the number of enterprises producing food category *j* in the province, and *M* is the total number of food enterprises in the province.

When the random inspection failure rate equals 0%, it would imply that no food safety issues exist and, consequently, that supervised inspections are unnecessary. In reality, however, food safety risks are always present, making a zero random failure rate highly unlikely. By contrast, if supervised inspections fail to detect problematic products or firms, the supervised inspection failure rate may be zero. Therefore, when the supervised inspection failure rate is less than or equal to the random inspection failure rate (i.e., supervision efficiency ≤ 1), this indicates that supervised inspections perform no better than random inspections. In other words, compared with random sampling, supervised inspections do not identify additional problematic products or firms. Conversely, when the supervised inspection failure rate exceeds the random inspection failure rate (i.e., supervision efficiency >1), it suggests that supervised inspections follow a problem-oriented approach and are more effective in detecting unsafe products and non-compliant firms. Accordingly, the ratio of the supervised inspection failure rate to the random inspection failure rate provides a relatively objective and reliable measure of the efficiency of food safety supervision inspections.

## References

[1] Guo Z Bai L Gong S. Government regulations and voluntary certifications in food safety in China: a review. Trends Food Sci Technol. (2019) 90:160–5. doi: 10.1016/j.tifs.2019.04.014

[2] Grace D. Food safety in low and middle income countries. Int J Environ Res Public Health. (2015) 12:10490–507. doi: 10.3390/ijerph120910490 26343693 PMC4586623

[3] Jin Y Zhou J. Food safety, sampling inspection and optimization of regulatory resource allocation: evidence from China's aquatic food inspection. Food Policy. (2026) 138:102976. doi: 10.1016/j.foodpol.2025.102976

[4] Han G Zhai Y. Perceptions of food safety, access to information, and political trust in China. Chinese J Commun. (2022) 15:534–57. doi: 10.1080/17544750.2022.2052129

[5] Yi Y Bremer P Mather D Mirosa M. Factors affecting the diffusion of traceability practices in an imported fresh produce supply chain in China. Br Food J. (2022) 124:1350–64. doi: 10.1108/BFJ-03-2021-0227

[6] Zhou J Jin Y Wang Y Liang Q. Do producers respond to quality information disclosure? The HACCP certification in meat industry. China Agric Econ Rev. (2022) 14:47–63. doi: 10.1108/CAER-06-2020-0156

[7] Zhao T Li T Liu D Luo Y. Influential factors and interventions for repeated production violations in food enterprises-Empirical evidence from China. J Clean Prod. (2024) 447:141421. doi: 10.1016/j.jclepro.2024.141421

[8] Qin K Zhang J Qian H Wu L. Risk evaluation, spatiotemporal evolution, and driving factors of provincial food safety in China. Ecol Indic. (2024) 166:112505. doi: 10.1016/j.ecolind.2024.112505

[9] Henson S Caswell J. Food safety regulation: an overview of contemporary issues. Food Policy. (1999) 24:589–603. doi: 10.1016/S0306-9192(99)00072-X

[10] Zhang B Lin J Liu R. Factors affecting the food firm's intention to control quality safety in China: the moderating effect of government regulation. Chinese Manag Stud. (2016) 10:256–71. doi: 10.1108/CMS-08-2015-0179

[11] Fernando Y Ng HH Yusoff Y. Activities, motives and external factors influencing food safety management system adoption in Malaysia. Food Control. (2014) 41:69–75. doi: 10.1016/j.foodcont.2013.12.032

[12] Deng H Kong L. Institutional characteristic, social co-governance and the effectiveness of food safety certification. Issues Agric Econ. (2022) 4:27–37.

[13] Li K Yin S Chen Y. Analysis of cross-regional transfer of food safety risks and its influencing factors—an empirical study of five provinces in East China. Foods. (2023) 12:1596. doi: 10.3390/foods12081596 37107391 PMC10138065

[14] Li T Zhao T Luo Y. Urbanization development, regulatory effort and food safety. J Macro-Qual Res. (2023) 11:117–28.

[15] Wang L Demeritt D Rothstein H. “Carrying the black pot”: food safety and risk in China's reactive regulatory state. Regul Gov. (2023) 17:469–90. doi: 10.1111/rego.12467

[16] Zhang H Lv J. Regional disparity and distribution dynamic evolution of food safety risk—an empirical study based on Dagum Gini Coefficient Decomposition and Kernel Density. J Public Manag. (2019) 16:77–88, 172–3.

[17] Yang Y Yu G Pan J Kreps GL. Public trust in sources and channels on judgment accuracy in food safety misinformation with the moderation effect of self-affirmation: evidence from the HINTS-China database. World Med Health Policy. (2023) 15:148–62. doi: 10.1002/wmh3.544

[18] Gao H Dai X Wu L Zhang J Hu W. Food safety risk behavior and social Co-governance in the food supply chain. Food Control. (2023) 152:109832. doi: 10.1016/j.foodcont.2023.109832

[19] Awuchi CG. HACCP quality, and food safety management in food and agricultural systems. Cogent Food Agric. (2023) 9:2176280. doi: 10.1080/23311932.2023.2176280

[20] Njunina V. HACCP Principles- What are the Steps of HACCP? Food Docs (2022). Available online at: https://www.fooddocs.com/post/haccp-principles (Accessed August 24, 2022).

[21] Nie W Liu C. Assessing food safety risks based on a geospatial analysis: toward a cross-regional food safety management. J Sci Food Agric. (2023) 103:6654–63. doi: 10.1002/jsfa.12761 37261721

[22] Tan BC Sarwar A Khan N Lau TC. The effects of consumer consciousness, food safety concern and healthy lifestyle on attitudes toward eating “green”. Br Food J. (2022) 124:1187–203. doi: 10.1108/BFJ-01-2021-0005

[23] Li Q Hu H Ma L Wang Z Arici M Li D . Evaluation of energy-saving retrofits for sunspace of rural residential buildings based on orthogonal experiment and entropy weight method. Energy Sustain Dev. (2022) 70:569–80. doi: 10.1016/j.esd.2022.09.007

[24] Dagum C. A new approach to the decomposition of the Gini income inequality ratio. Empir Econ. (1997) 22:515–31. doi: 10.1007/BF01205777

[25] Carta JA Ramirez P Bueno C. A joint probability density function of wind speed and direction for wind energy analysis. Energy Convers Manag. (2008) 49:1309–20. doi: 10.1016/j.enconman.2008.01.010

[26] Liu F Rhim H Park K Xu J Lo CK. HACCP certification in food industry: trade-offs in product safety and firm performance. Int J Prod Econ. (2021) 231:107838. doi: 10.1016/j.ijpe.2020.107838

[27] Lu D Lin Y Le S Chen Y Feng C Qian Z . Assessment of POPs in foods from western China: machine learning insights into risk and contamination drivers. Environ Int. (2025) 199:109458. doi: 10.1016/j.envint.2025.109458 40250238

[28] Xiao X Gao Y. An event study of the effects of regulatory changes on the food industry: the case of the Food Safety Law of China. China Agric Econ Rev. (2017) 9:81–92. doi: 10.1108/CAER-01-2014-0006

[29] Tao Z Zhang Z Shangkun L. Digital economy, entrepreneurship, and high-quality economic development: empirical evidence from urban China. Front Econ China. (2022) 17:393.

[30] Chen YQ Chen YH. Economic growth, income inequality and food safety risk. Foods. (2023) 12:3066. doi: 10.3390/foods12163066 37628065 PMC10453881

[31] Focker M Van Der Fels-Klerx HJ. Economics applied to food safety. Curr Opin Food Sci. (2020) 36:18–23. doi: 10.1016/j.cofs.2020.10.018

[32] Dong Q Zhong K Liao Y Xiong R Wang F Pang M. Coupling coordination degree of environment, energy, and economic growth in resource-based provinces of China. Res Policy. (2023) 81:103308. doi: 10.1016/j.resourpol.2023.103308

[33] Wei M Li S. Study on the measurement of economic High-Quality development level in China in the new era. J Quant Technol Econ. (2018) 35:3–20.

[34] Kakwani N Wang X Xue N Zhan P. Growth and common prosperity in China. China World Econ. (2022) 30:28–57. doi: 10.1111/cwe.12401

[35] Li T Bo H. Quantitative evaluation of supervision sampling efficiency of food safety in China. Sci Technol Food Indus. (2022) 43:301–10. doi: 10.13386/j.issn1002-0306.2021100174

[36] Jin C Levi R Liang Q Renegar N Springs S Zhou J . Testing at the source: analytics-enabled risk-based sampling of food supply chains in China. Manage Sci. (2021) 67:2985–2996. doi: 10.1287/mnsc.2020.3839

